# Swallow–Breath Interaction and Phase of Respiration with Swallow during Non-Nutritive Suck in Infants Affected by Neonatal Abstinence Syndrome

**DOI:** 10.3389/fped.2017.00214

**Published:** 2017-10-04

**Authors:** Eric W. Reynolds, Debbie Grider, Cynthia S. Bell

**Affiliations:** ^1^Division of Neonatology, Department of Pediatrics, McGovern Medical School, University of Texas Health Science Center at Houston, Houston, TX, United States; ^2^Division of Neonatology, Department of Pediatrics, University of Kentucky, Lexington, KY, United States; ^3^Department of Pediatrics, McGovern Medical School, University of Texas Health Science Center at Houston, Houston, TX, United States

**Keywords:** neonatal abstinence syndrome, non-nutritive suck, suck–swallow–breath coordination, infant feeding, neonatology

## Abstract

**Background:**

The development of suck–swallow–breath rhythms during non-nutritive suck (NNS) may be an indicator of neurologic integrity. We have described swallow–breath (SwBr) interaction and phase of respiration (POR) with swallow during NNS in low-risk preterm (LRP) infants. NNS in infants with neonatal abstinence syndrome (NAS) has not been described with our method.

**Method:**

Suckle, swallow, thoracic motion, and nasal airflow were measured during NNS in 10 infants with NAS and 12 unaffected infants (control). Logistic regression models were fit to describe the three types of SwBr and five types of POR in terms of the independent variables (gender, gestational age, birth weight, postmenstrual age, weeks postfirst nipple feed and swallows per study). We also compared the NAS group to 16 LRP infants.

**Results:**

In the NAS group, there were 94 swallows in 18 studies. In the control group, there were 94 swallows in 12 studies. There were statistical differences between groups for all three types of SwBr. The distribution of SwBr in NAS was similar to LRP infants with NAS having fewer swallows with attenuated respiration and more with central apnea. For POR, there were few differences. Over time, the distribution of SwBr in NAS infants approaches that of control infants.

**Discussion:**

Variability in SwBr and POR during NNS may represent neurologic dysfunction in infants with NAS. Specifically, term infants with NAS display an immature pattern of SwBr making them more similar to preterm infants, rather than a unique pathology. The distribution of SwBr and POR in NAS infants becomes more like term infants, possibly representing catch-up development as the NAS symptoms resolve.

**Conclusion:**

SwBr in babies with NAS is different from that of unaffected term infants, actually being similar to preterm infants. Infants with NAS exhibit a dysmature pattern of NNS development which resolves over time.

## Introduction

Efficient suckle-feeding can be considered to be the most complex skill a newborn infant must master to attain independent survival. However, feeding problems are frequent in preterm infants (1) and can lead to prolonged hospital stays (2). Poor feeding in the neonatal period may be an early indicator of neurologic injury (3, 4) and has been linked to language delay later in life (5).

The development of efficient suckle-feeding is dependent on the maturation and coordination of neuronal central pattern generators controlling suck, swallow, and breath (SwBr) (6). These same central pattern generators are also activated during non-nutritive suck (NNS). Thus, NNS may provide an earlier marker of intact neurodevelopment than nutritive feeding does.

We have previously used our method to study NNS in low-risk preterm (LRP) infants (7) and have shown that the interaction of SwBr and the phase of respiration (POR) incident to swallow develop in a predictable pattern in these infants. The progression of SwBr is influenced by increasing opportunities to practice the skill, or what can be considered “learning.” The progression of POR was more affected by measures of maturation, indicating a developmental progression. Our method has not yet been applied to pathologic conditions.

Neonatal abstinence syndrome (NAS) is a constellation of withdrawal-like symptoms experienced by infants born to mothers who have been chronically taking opiates or other drugs/medications during the pregnancy. Feeding difficulty and disruption of suck–swallow–breath rhythms have been reported in infants affected by NAS (8, 9). In general, abnormal sucking and feeding behaviors in infants affected by NAS include excessive sucking, inattention, fussiness, and decreased swallow efficacy. The specifics of suck–swallow–breath organization during NNS in infants affected by NAS have not been described.

## Materials and Methods

We performed a prospective observational study comparing suck–swallow–breath coordination in infants with NAS and unaffected infants. The study participants included 10 term infants with NAS and 12 healthy term (control) infants. We also included a group of 16 LRP infants who were concurrently enrolled in the study and described in a previous manuscript (7). Table 1 is a list of the abbreviations provided to help the reader understand the presented information.

**Table 1:** Abbreviations and acronyms used in this study.

NAS	Neonatal abstinence syndrome	Group of symptoms experienced by infants as a consequence of opioid withdraw. The group of infants in this study who were diagnosed and treated for neonatal abstinence syndrome
NAS1st	Subgroup of NAS study group	Subgroup of NAS infants including only one study from each patient. Allows analysis without repeated measures
LRP	Low-risk preterm	Group of “healthy” preterm infants with no sepsis, no IVH and relative low-risk for the development of bronchopulmonary dysplasia
NNS	Non-nutritive suck	Act of infant sucking on a pacifier with no milk intake
SwBr	Swallow–breath interaction	How swallow interacts with breath. Can occur in 3 types (AR, OA, CA).
AR	Attenuated Respiration	Deflection of the slope of the nasal airflow tracing without interruption in the overall breathing rhythm
OA	Obstructive Apnea	Cessation of nasal airflow for the duration of a swallow with continued chest movement
CA	Central Apnea	Cessation of both nasal airflow and chest movement for the duration of a swallow
POR	Phase of Respiration Incident to Swallow	Where in the respiratory cycle a swallow occurs. Can occur in 5 types (BE, ME, EE, MI, AP)
BE	Beginning Expiration	Transition from inspiration to expiration
ME	Mid-Expiration	Point between beginning expiration and end expiration
EE	End Expiration	Transition from expiration to inspiration
MI	Mid-Inspiration	Point between end expiration and beginning expiration
AP	Apnea	Period of no discernable breathing for 1 s prior to the time of a swallow

The 10 infants in the NAS group were diagnosed by a combination of *in utero* exposure to substances known to result in NAS and postnatal symptoms consistent with the diagnosis. *In utero* exposures included benzodiazipines, methadone, cocaine, oxycotin, klonipin, fentanyl, THC, oxycodone, and buprenorphine. Five infants had poly-drug exposure. Five infants had single drug exposures (four methadone and one oxycodone). These included four mothers who were compliant with a methadone treatment program and another mother who was taking chronic pain medication. Finnegan scores at the time of enrollment ranged from 9 to 20. In our NICU at the time, babies who were at risk for NAS were monitored for symptoms of NAS and scored with the standard Finnegan Score tool (10). Per institutional norm, infants who score 8 or more on 2 consecutive scores, or 12 or more once, were given a diagnosis of NAS and treated with opiate replacement therapy. At the time of this study, the treatment for NAS in our facility was not standardized. Infants were treated with morphine or methadone. The protocol for adjusting doses was not proscribed. Two infants required adjunctive therapy with phenobarbital. Infants were studied once per week from the time of enrollment until discharge from the hospital. There were 94 swallows collected from 18 studies in this group.

The control group included 12 babies born between 37 and 42 weeks of gestation, appropriate size for gestational age, 5-min Apgar score of 7 or more and with no congenital anomalies or metabolic disorders. These infants were studied once prior to discharge from the hospital. There were 94 individual swallow events collected over 12 studies from these infants.

We also compared the NAS group to a group of 16 LRP infants who were born at less than 36 weeks (mean gestational age at birth: 28 5/7, mean postmenstrual age at study: 35 3/7), appropriate size for gestational age (mean birth weight: 1,056), no congenital anomalies, no IVH of grade 3 or 4 and deemed to be “low risk” for bronchopulmonary dysplasia per the definition in our previous publication (7). There were 176 swallows in 35 studies in this group.

Because of potential statistical problems with repeated studies in the NAS group and not in control infants, we analyzed the data using only a single study for each NAS infant. This decreased the number of studies in this group (NAS1st) to 10 and the number of swallows to 49.

Informed consent was obtained from the parent(s) of each infant prior to the infant’s participation in the study. The project complies with all applicable HIPAA standards and was approved by the Institutional Review Board of the University of Kentucky.

We have previously published the specific method and equipment for preparing the babies for the study and data collection (7). To summarize the salient portion of the study for this project, the infant study participants were prepared in the following manner:

- A 5 F nasopharyngeal catheter was placed and connected to a pressure transducer to measure swallow pressure.
- A second catheter was placed through a pacifier so that the catheter tip was flush with the nipple and connected to a transducer to measure suckle pressure.
- Respiratory effort was measured with a stretchable band placed around the infant’s chest.
- Nasal airflow was measured with a thermistor bead placed at the opening of the nares.

The infants were offered a pacifier for 1-min of NNS just prior to a nutritive feeding. Data were collected and displayed as multichannel linear graphs, using the Windaq Waveform Browser (Dataq Industries, Akron, OH, USA). The entire 1-min sequence of NNS was canvassed for swallows, noted as deflections in the naso-pharyngeal pressure recording. The type of SwBr and POR were classified, as described below. During NNS, swallow occurs infrequently. When it does occur, the SwBr must abruptly interact for about 1–2 s, in what has been termed “deglutition apnea” (11), and should not be confused with apnea (AP) of prematurity which requires 15–20 s of no breathing to be defined as AP.

**Figure 1:**
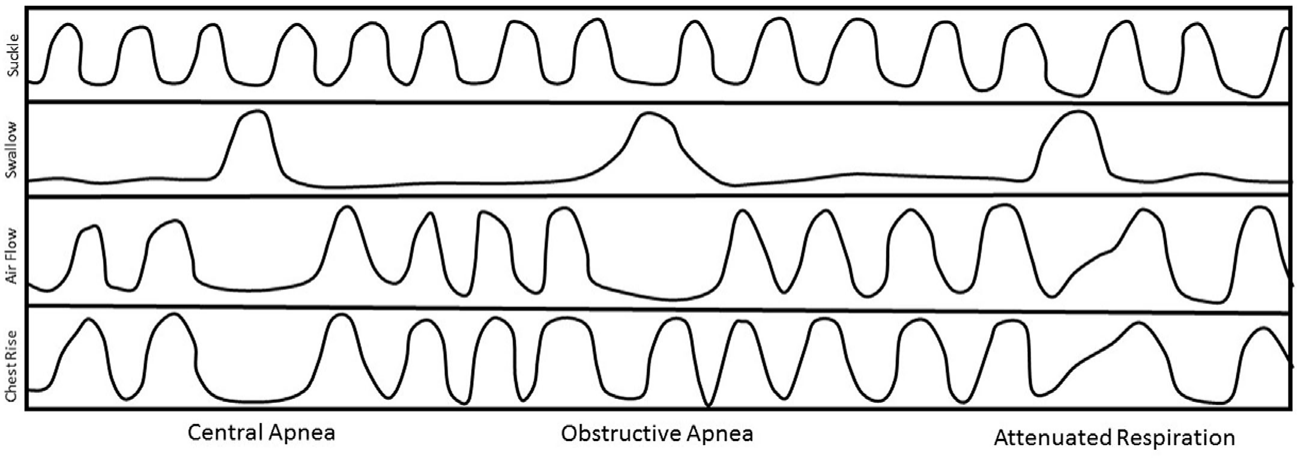
Stylized examples of each type of swallow and breath (SwBr). Example of a four-channel recording of suckle, swallow, air flow, and chest movement. Three swallows are shown as deflections in the swallow channel tracing. SwBr is defined by the respiratory effort and airflow coincident to each swallow.

We can identify three types of SwBr interaction: central apnea (CA) (cessation of both nasal airflow and chest movement), obstructive apnea (OA) (cessation of nasal airflow but continued rhythmic chest movement), or attenuated respiration (AR) (a slight deflection of the slope of the respiratory line on the graph at the time of the swallow without disruption of the respiratory rhythm). Examples of each type of SwBr are shown in Figure 1.

The respiratory cycle can be divided into five phases (POR): beginning expiration (BE), mid-expiration (ME), end-expiration (EE), mid-inspiration (MI), and AP. Swallows occur at any of these PORs. It has been hypothesized that the most mature pattern exists when the swallow occurs at a point of minimal air movement (BE, EE, or AP) (11).

Independent variables for this analysis included gender, birth weight, gestational age, postmenstrual age, number of swallows in the study, and weeks postfirst nipple feed (time between first nipple feed and day of study). Day-of-life at the time of the study is used only for demographic descriptions and not for analysis because it would create a collinearity issue since postmenstrual age is a function of gestational age + day-of-life.

Infants were invited to return for developmental testing [Bayley-III and Preschool Language Scales version IV (PLS-IV)] at 12 and 24 months. 11 infants from the control group returned at 12 and 24 months. 7 infants in the NAS group returned at 12 months and 5 returned at 24 months. One of the NAS infants at 24 months could not complete all of the assessments.

We used Stata 13.1 (College Station, TX, USA) to calculate descriptive statistics on patient demographics and to construct logistic regression models relating the odds of each type of SwBr and POR to the independent variables defined above. Statistical inferences were made *via* generalized estimating equations (12) with an exchangeable structure to take into account the correlations inherent to repeated assessments on the same baby. The number of patients/studies came from a subset of a larger study of infant feeding. Power calculations were based on that study and these patients were selected because they had data that was useable for this analysis.

## Results

### Demographics

Demographic characteristics of the infants in the study groups are shown in Table 2. Each group was approximately 60% female and 40% male. Mean birth weight and gestational age were similar for each group. Babies in the NAS group were studied weekly during their in-hospital treatment. Thus, day-of-life, postmenstrual age, and weeks postfirst nipple feed are greater in the NAS group. The number of swallows per study was not different between the two groups. Because of the bias caused by having repeated studies in the NAS group and not in the control group, we performed the analysis using only a single study for each baby in the NAS group (NAS1st). The significant differences for day-of-life and weeks postfirst nipple feed remained. This is because babies in the NAS group were enrolled in the study after their symptoms had progressed to the point of being diagnosed with NAS, which can take over a week, depending on the drug of exposure, timing of the last dose and specific genetic polymorphisms which affect NAS timing and severity. The difference in Postmenstrual Age was eliminated.

**Table 2:** Patient population characteristics.

	Control	NAS	NAS1st
Swallows	94	94	49
Studies	12	18	10
Babies	12	10	10
Day-of-life	2.8 + 1*	17 + 11*	8.7 + 3.2*
Postmenstrual age	39.8 + 0.9*	41.7 + 2.4*	40.2 + 1.6*
Weeks postfirst nipple feed	0.3 + 0.2*	2.4 + 1.7*	1.2 + 0.4*
Swallows per study	8 + 7	5 + 2	5 + 2
Gestational age	39.5 + 1.0	39.5 + 1.8	
Birth weight	3,344 + 393	3,045 + 560	
Male/female	5/7 (42/58%)	4/6 (40/60%)	

*There are significant differences between NAS and control for day-of-life (*p* < 0.0001), postmenstrual age (*p* = 0.003), and weeks postfirst nipple feed (*p* < 0.0001). There are significant differences between NAS1st and control for day-of-life (*p* < 0.0001) and weeks postfirst nipple feed (*p* < 0.0001)*.

*indicates statistical significance.

### Distribution of SwBr and POR in NAS

The percent of swallows occurring at each type of SwBr and POR for the study groups are shown in Table 3. Section A shows the results of univariate analysis comparing NAS and control with differences for SwBr as follows. AR is less common in NAS than control (OR = 4.05, *p* = 0.0041). There was no difference for OA. CA was more common in NAS than control (OR = 0.212, *p* = 0.0353). For POR, there were no differences between NAS and control infants for BE, ME, EE, or AP. MI was more common in NAS than control (OR = 0.28, *p* = 0.0361).

**Table 3:** Distributions of SwBr and POR for each group.

	SwBr			POR				
	CA	OA	AR	BE	ME	EE	MI	AP
**A**								

Control	5	20	74	41	19	24	5	10
NAS	22	36	41	28	12	28	19	14
	*		*				*	

**B**								

Control	3	11	91	68	24	21	18	0
NAS	19	27	57	32	17	26	32	3
	*	*	*	*				*

**C**								

Control	5	20	74	41	19	24	5	10
NAS1S	35	27	39	22	14	39	4	20
	*		*	*				

**D**								

NAS	22	36	41	28	12	28	19	14
LRP	25	32	43	21	9	48	7	15
						*		

*A: distribution of SwBr and POR for control vs. NAS. There are significant differences for CA and AR in SwBr and for MI in POR*.

*B: predicted distribution of SwBr and POR with multivariate analysis. There are significant differences for all three types of SwBr and BE and AP in POR*.

*C: distribution of SwBr and POR for control vs. NAS1st. There are significant differences for CA and AR in SwBr and for BE in POR*.

*D: distribution of SwBr and POR for NAS vs. LRP. There are no significant differences for SwBr. There is a significant difference for EE in POR*.

*indicates statistical significance.

Using multivariate analysis (Table 3, B) to control for differences in the independent variables, CA was predicted to be more common in NAS then control (OR = 0.1117, *p* = 0.0075). OA was more common in NAS then control (OR = 0.351, *p* = 0.0241). AR was less common in NAS than control (OR = 8.1, *p* = 0.0004). OA was more common in females (OR = 0.387, *p* = 0.0011) and AR was more common with advancing weeks postfirst nipple feed (OR = 1.64 per week, *p* = 0.0210). There were no predicted differences between NAS and control for ME, MI, or EE. BE was less common in NAS than control (OR = 4.60, *p* = 0.0026). AP was more likely in NAS than control (OR = 0.099, *p* = 0.0092). AP was less common with advancing weeks postfirst nipple feed (OR = 0.456 per week, *p* = 0.0115).

### SwBr and POR with a Single NAS Study

Babies in the control group underwent one study prior to discharge from the hospital, while babies in the NAS group were studied weekly during their hospital stay. In order to evaluate the data without the inherent bias introduced by repeated measures on the same baby, the analysis was performed using only a single study from each baby in the NAS group (NAS1st). This decreased the number of swallows for analysis to 49. Table 3 shows the results of univariate analysis of NAS1st vs. control. The statistical difference between NAS1st and control for CA and AR remained. CA was more common in NAS1st than control infants (OR = 0.146, *p* = 0.0042). AR was less common in NAS1st than control infants (OR = 4.401, *p* = 0.0022). For POR, the significant difference initially noted between NAS1st and control for MI was no longer present. However, BE was less common in NAS1st than control infants (OR = 2.565, *p* = 0.0451).

### SwBr and POR in NAS and LRP

Having established that the distribution of SwBr is different between NAS and control, we were interested to determine if the NAS infants were similar to preterm infants. Thus, we compared the SwBr in NAS to a group of LRP infants. As shown on Table 3, the percentages of each type of SwBr were similar to that of the LRP babies, with no statistically significant differences for any SwBr. There were some differences noted for POR.

### Developmental Follow-up

Study participants returned for developmental follow-up assessments with the Bayley Scales of Infant Development version III (Bayley-III) and the PLS-IV. Table 4 shows the average scores for infants in the control and NAS groups. At 12-month follow-up, the average scores for Bayley-III and PLS-IV assessments were not different between the control and NAS infants. At 24 months, Bayley-III scores were not different between control and NAS infants. PLS-IV scores at 24 months were statistically lower in the NAS group, but the number lost to follow-up limits the ability draw conclusions from this data.

**Table 4:** Developmental follow-up of infants in control and NAS groups.

		Control	NAS	*p*-Value
**12 months**				

	Enrolled	12	10	
	Follow-up	11	7	
Bayley-III	Cognitive	102.5 ± 10.1	102.9 ± 12.2	0.9524
	Motor	96.2 ± 5.5	97.4 ± 15.3	0.8512
	AC	99.6 ± 9.5	102.7 ± 20.8	0.7367
PLS-IV	EC	103.8 ± 18.4	104.4 ± 16.1	0.9458
	TLS	102.3 ± 14.4	103.3 ± 18.5	0.9079

**24 months**				

	Follow-up	11	5^a^	
Bayley-III	Cognitive	95.3 ± 6.3	90.7 ± 5.2	0.1477
	Motor	99.7 ± 9.1	97 ± 5.7	0.4899
	AC	101.8 ± 12.6	82.2 ± 11.2	**0.0205**
PLS-IV	EC	105.5 ± 12.6	79.8 ± 12.1	**0.0081**
	TLS	104 ± 13.7	82.2 ± 12.1	**0.0086**

*There were statistical differences for PLS scores at 24 months*.

*^a^One infant in the NAS group at 24 months did not complete all of the assessments*.

*AC, auditory comprehension; EC, expressive communication; TLS, total language score*.

*Boldface indicates statistical significance*.

## Discussion

Successful newborn feeding requires coordination of the rhythms of suck, SwBr (13–16). Feeding problems have been identified as a sequelae of hypoxic–ischemic injury (17) and later neurologic injury such as cerebral palsy (16). Mild disruptions of the rhythmicity of suckle feeding can be linked to less severe injury (18) and may predict subsequent feeding and neurologic problems (19, 20). Abnormal newborn feeding behaviors have been linked to language delay at 18 months (5). The progression of normal rhythmic suckle feeding is dependent on the interaction of brainstem central pattern generators for suck, SwBr (6). These central pattern generators are also activated during NNS. Therefore, we hypothesize that the organization of suck, SwBr during NNS may be an early indication of the integrity of the neonatal CNS.

Babies affected by NAS are known to have disorders of the central nervous system which can manifest as excessive suck, abnormal breathing and feeding difficulty. Kamal et al. has shown that infants affected by NAS have an abnormal ventilatory response to hypercarbia in the newborn period and at 6–12 weeks of life that may increase their risk for SIDS (21, 22). Finnegan et al. included excessive suck, respiratory distress and other gastrointestinal disturbance among the symptoms of NAS included in their tool to track the severity of NAS disease (10). Most studies of abnormal feeding related to NAS have focused on behaviors of the infant or mother. Maguire et al. (23) found the infants with NAS exhibit characteristic behaviors such as tremors, hyperextension, altered muscle tone, and multiple transitions between behavioral states that contribute to difficult feeding. Furthermore, these infants spend an excessive amount of time fussing, including averting the face from the mother, pulling or turning away or otherwise resisting, hyperextension, flailing arms or vocal objections to feeding, “but not a robust cry.” LaGasse et al. (8) found that opiate-exposed infants had prolonged sucking with fewer pauses, more feeding problems such as spitting up and refusal, and increased arousal. None of these studies looked at the development of suck–swallow–breath rhythms. Gewolb et al. (9) evaluated the integration of these rhythms during feeding in infants born to mothers with drug-abuse problems. They found subtle abnormalities of respiratory control and swallow rhythmicity in drug-exposed infants. Drug-exposed infants were less efficient feeders than control infants (decreased volume/swallow), but this decreased efficiency was offset by a faster swallow rate. The differences they noted at 3 days of life between drug-exposed and unaffected control infants were not present when the infants were studied at 1 month of life.

Very few studies of NAS have focused on NNS and none have looked at the integration of SwBr during NNS. Maone et al. (24) offered cocaine-exposed infants, and control babies, a standard pacifier and a sucrose flavored pacifier. The drug-exposed cohort had about the same suck rate as control infants when offered the unflavored pacifier. However, when offered a sucrose-flavored pacifier, the cocaine-exposed cohort increased their suckle rate in a statistically significant manner.

Our previous work evaluating NNS in LRP infants supports the idea that there is an interaction between SwBr that occurs during NNS, similar to deglutition AP during nutritive feeding and that this interaction, which we identified as SwBr and POR incident to swallow, or POR, progresses in a predictable and measurable fashion (7). We hypothesized that disruptions of this progression may correlate with abnormalities of central nervous system development. Now, we turn our attention to term infants and a disease state that is known to cause feeding difficulty, suck irregularities and neurologic abnormalities.

In our primary analysis, we compared a group of term infants affected by NAS to a group of healthy infants delivered at term gestation. We found particularly pronounced differences between the infants with NAS and control infants, especially related to SwBr. The differences were less pronounced for POR. Babies with NAS had significantly more swallows occurring with CA and fewer occurring with AR than control babies. There was a nonsignificant trend toward more swallows with OA in the NAS group as well. When controlling for identified confounders with multivariate analysis, the differences in predicted distributions for CA and AR were still present. Additionally, the difference in predicted percentage of swallows at OA became statistically significant.

The only significant difference for POR was for more swallows occurring at MI. It may be more difficult to identify statistically significant differences in POR since there are more categories in which swallows are grouped, thus requiring more statistical power. Furthermore, in our previous study of LRP infants, we found that the progression of POR was affected mostly by postmenstrual age, suggesting a developmental process that was not affected by practice or learning (7). Since there is very little difference in the postmenstrual age for babies across this study, it is not surprising that we find only minor differences in POR.

Given that bias can be introduced into the analysis by the fact that some babies in the NAS group were studied weekly while in the hospital but control babies only had a single study, we sought to limit the effect of repeated measures by performing the analysis with a single study for each NAS baby. This decreased the number of swallows available for analysis and thus decreased the statistical power. However, the statistically significant differences for SwBr remained with more swallows from NAS infants occurring at CA and fewer occurring at AR. The difference noted in POR for MI was no longer present, but a statistical difference for BE was noted. It is possible that either, or both, of these associations may occur by chance, given the number of comparisons we are analyzing in this work. Even if these differences in the distribution of POR are real, it is easy to conclude that any variability in POR introduced by NAS is quite small compared to the differences in SwBr.

Having established that the distribution of SwBr is different for infants with NAS versus control infants, we were interested to determine if these differences were a reflection of immaturity of the suck–swallow–breath reflexes or a completely different pathological process. Thus, we compared the NAS infants to a group of LRP infants. These infants were relatively healthy aside from being born preterm, with no sepsis, no severe intraventricular hemorrhage and at low-risk for developing bronchopulmonary dysplasia. These babies were studied once per week from the onset of nipple feeding until discharge from the NICU. Interestingly, there were no statistically significant differences between the NAS and LRP groups for any of the SwBr types. There was a single significant difference in POR noted, with more swallows occurring at EE in the LRP group. Thus, it appears that the coordination of suck–swallow–breath rhythms in infants with NAS is immature, more like preterm infants, when compared to term infants.

Our previous work in preterm infants suggested that the distribution of SwBr was heavily influenced by the number of attempts the baby had to try nipple feeding, regardless of Gestational Age at delivery or Postmenstrual Age at the time of the study (7). Thus, it seems that the coordination of SwBr may be a learnable skill. Since babies with NAS do experience improved feeding over the course of their treatment, it is logical to assume that there will be an improvement in their SwBr distribution. In fact, comparing the distribution of SwBr in the total NAS group and the single-study NAS group, one can appreciate that the additional NAS studies are changing the SwBr distribution toward that of term infants. That is to say, the percentage of CA in NAS1st is 35%. With the added weekly studies, the percentage of CA is 22% in the NAS group. For control infants it is 5%. Similarly, AR changes from 39% in NAS1st to 57% in whole NAS group and 74% in control infants. Thus, we hypothesize that the distribution of SwBr in these NAS infants is approaching that of the control infants in a manner similar to that described by Gewolb et al. (9) for nutritive feeding. This also suggests that the differences noted in day-of-life, postmenstrual age, and weeks postfirst nipple feed does not account for the statistical differences in our results. Since NAS babies are older (day-of-life, postmenstrual age) and have been feeding longer (weeks postfirst nipple feed) we would predict that they would be more mature than the control group, rather than the immature pattern we have described.

Previous studies of feeding in infants with NAS have described excessive fussiness, inattention, spitting, and decreased efficiency. It is possible that the pathologic immaturity of suck–swallow–breath reflexes is contributing to the infants’ overall state of dysfunction. It is unclear from this data if the immature suck–swallow–breath coordination we are describing is a function of *in utero* exposure to various toxins or occurs as a consequence of the treatment for NAS. This dataset does not allow us to answer these questions because there were variations in the specific exposures and the choice of treatment as well as the fact that all of the NAS babies were receiving medical treatment at the time of the study. If we will see a similar dysfunction with studies of nutritive feeding is also unclear.

## Conclusion

Infants affected by NAS have an immature pattern of SwBr during NNS when compared with healthy term infants, making them more like preterm infants in this respect. There is evidence to support the idea of “catch-up” development as the distribution of SwBr approaches that of healthy term infants as the affected infants are studied subsequently over time. This is interesting because it shows that infants with NAS are not affected by a unique pathology, but rather are showing an immature pattern which improves over time. It is unclear if the dysfunction of SwBr is a consequence of the initial exposure or due to the treatment. Likewise, the improvement in SwBr may be simply related to maturation or could be due to receiving appropriate treatment. The immature pattern of suck–swallow–breath reflexes may be a contributing factor to the overall feeding difficulty experienced by babies with NAS. Future studies can focus on improving the development of suck–swallow–breath integration in infants with NAS.

## Ethics Statement

Informed consent was obtained from the parent(s) of each infant prior to the infant’s participation in the study. The project complies with all applicable HIPAA standards and was approved by the Institutional Review Board of the University of Kentucky.

## Author Contributions

ER was responsible for the project design, data collection and interpretation, and manuscript preparation. DG was responsible for subject identification and enrollment and data collection. CB was responsible for statistical methods, data analysis, and manuscript preparation.

## Conflict of Interest Statement

The authors declare that the research was conducted in the absence of any commercial or financial relationships that could be construed as a potential conflict of interest.
